# MALT1 inhibitor MI-2 induces ferroptosis by direct targeting of GPX4

**DOI:** 10.1073/pnas.2507997122

**Published:** 2025-05-09

**Authors:** Eikan Mishima, Thomas J. O’Neill, Kai P. Hoefig, Deng Chen, Gesine Behrens, Bernhard Henkelmann, Junya Ito, Kiyotaka Nakagawa, Vigo Heissmeyer, Marcus Conrad, Daniel Krappmann

**Affiliations:** ^a^Institute of Metabolism and Cell Death, Molecular Targets and Therapeutics Center, Helmholtz Munich, Neuherberg 85764, Germany; ^b^Department of Redox Molecular Medicine, Tohoku University Graduate School of Medicine, Sendai 980-8575, Japan; ^c^Research Unit Signaling and Translation, Group Signaling and Immunity, Molecular Targets and Therapeutics Center, Helmholtz Munich, Neuherberg 85764, Germany; ^d^Research Unit Molecular Immune Regulation, Molecular Targets and Therapeutics Center, Helmholtz Munich, Munich 81377, Germany; ^e^Laboratory of Food Function Analysis, Graduate School of Agricultural Science, Tohoku University, Sendai 980-8572, Japan; ^f^Institute for Immunology, Medical Faculty, Biomedical Center, Ludwig-Maximilians-Universität München, Planegg-Martinsried 82152, Germany; ^g^Translational Redox Biology, Natural School of Sciences, Technical University of Munich, Garching 85748, Germany; ^h^Faculty of Biology, Ludwig-Maximilians-Universität München, Planegg-Martinsried 82152, Germany

**Fig. 1.**
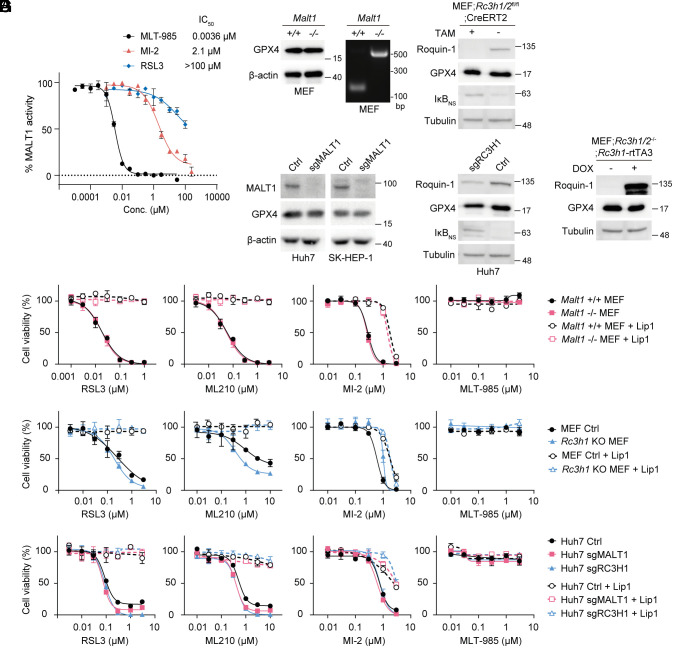
MI-2 induces ferroptosis independent of MALT1–Roquin-1. (*A*) Dose titration curves and IC_50_ values of recombinant GST-MALT1 (aa 325–760) activity after incubation with indicated compounds using the fluorescence substrate (Ac-LRSR-AMC) assay. (*B*) GPX4 amounts in *Malt1*^+/+^ and *Malt1*^−/−^ MEFs (*Right*: genotyping PCR for *Malt1*) or Roquin-1 KO induced by 4-hydroxy-tamoxifen (TAM) treatment of *Rc3h1/2^fl^*^/fl^;CreERT2 MEFs. (*C*) GPX4 amounts in Huh7 and SK-HEP-1 ctrl (Cas9-only), sgMALT1, and sgRC3H1 cells. (*D*) GPX4 amounts in *Rc3h1/2*^−/−^;rtTA3 MEFs after doxycycline (DOX)-induced reconstitution of Roquin-1; expression changes of Roquin-1 target IκB_NS_ to verify genetic manipulation. (*E*–*G*) Cell viability of indicated MALT1 and Roquin-1 KO cells (B-D) after GPX4 inhibitor (RSL3 or ML210), and MALT1 inhibitor (MI-2 or MLT-985) treatment with or without ferroptosis inhibitor liproxstatin-1 (Lip1, 1 μM) for 24 h. Data depict mean ± SD, n = 3 (*A* and *E*–*G*).

Ferroptosis, a form of cell death driven by excessive lipid peroxidation, is induced by inhibiting GPX4, a key regulator of ferroptosis. Wang et al. reported that MALT1 protease activity regulates GPX4 protein stability, thereby modulating sensitivity of cancer cells to ferroptosis (1). Their study suggests that MALT1 protects GPX4 from ubiquitin-dependent degradation by cleavage of the E3 ubiquitin ligase Roquin-1 (RC3H1). However, ferroptosis induction by MALT1 inhibition was investigated using MI-2, a low-potency, irreversible MALT1 inhibitor (IC_50_ 2.1 µM; Fig. 1*A*) (2). Here, we reexamine the role of the MALT1–Roquin-1 axis and unravel the actual ferroptosis-inducing mechanism of MI-2.

Neither MALT1 nor Roquin-1 knockout (KO) significantly influenced GPX4 expression in mouse embryonic fibroblasts (MEFs) or Huh7 and SK-HEP-1 liver cancer cells (Fig. 1 *B* and *C*). Furthermore, Roquin-1 reconstitution in KO MEFs had no impact on GPX4 expression (Fig. 1*D*). In MEFs and Huh7 cells, sensitivity to the bona fide GPX4 inhibitors RSL3 and ML210 remained unaltered in the absence of MALT1 or Roquin-1 (Fig. 1 *E*–*G*). These discrepancies may arise from different culture conditions, as GPX4 expression is influenced by medium components, particularly selenium concentrations (3). Nonetheless, as reported, MI-2 induced cell death, which was rescued by the ferroptosis inhibitor liproxstatin-1 (Lip1) [Fig. 1 *E*–*G* (1)]. However, ferroptosis induction by MI-2 was not impaired in MALT1- or Roquin-1-deficient cells. Moreover, the highly potent and selective MALT1 inhibitor MLT-985 (IC_50_ 3.6 nM, Fig. 1*A*) (4) failed to induce ferroptosis, demonstrating that MI-2 acts independently of the MALT1–Roquin-1 axis.

**Fig. 2.**
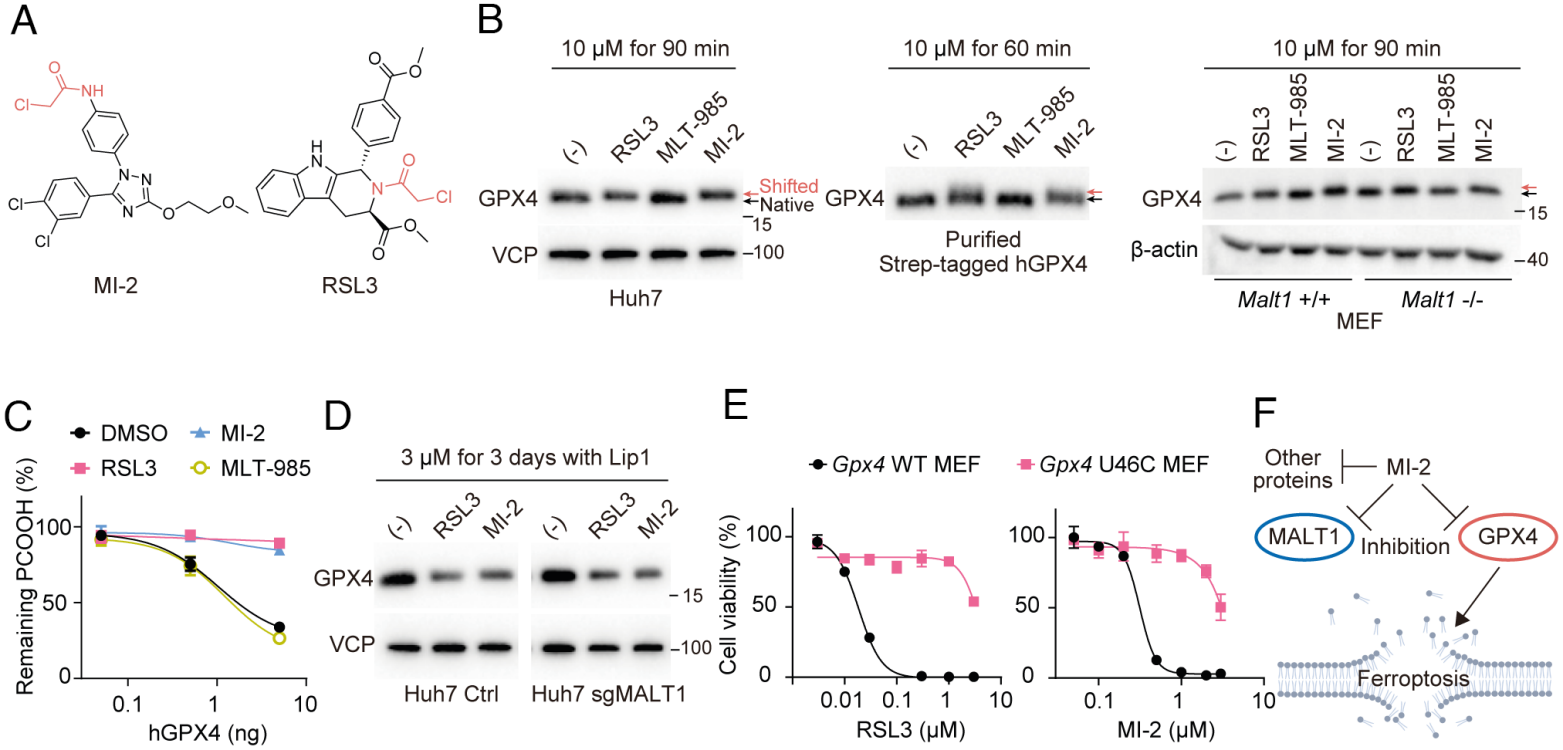
MI-2 directly binds and inhibits GPX4. (*A*) MI-2 and RSL3 chemical structures (red: chloroacetamide). (*B*) GPX4 migration-shift in Huh7 cells (*Left*), purified StrepII-tagged GPX4 protein (*Middle*), or *Malt1*^+/+^ and *Malt1*^−/−^ MEFs (*Right*) after incubation with indicated compounds. (*C*) Affinity-purified GPX4 from StrepII-tagged GPX4-expressing HT-1080 cells pretreated with indicated inhibitors (10 μM with Lip1) for 3 h was incubated with reduced glutathione (1 mM) and phosphatidylcholine hydroperoxide (PCOOH, 10 μM; 30 min at 37 °C). PCOOH was quantified by LC–MS. (*D*) GPX4 degradation analyzed by immunoblot after RSL3 and MI-2 treatment in MALT1 WT and KO Huh7 cells. (*E*) Cell viability of *Gpx4* WT and *Gpx4* U46C MEFs treated with RSL3 and MI-2 for 24 h. (*F*) Scheme for direct GPX4 inhibition and ferroptosis induction by MI-2 (created by BioRender.com). Data are mean ± SD, n = 3 (*C* and *E*).

MI-2 and RSL3 share a chloroacetamide moiety (Fig. 2*A*), an electrophilic warhead in RSL3 that irreversibly inactivates GPX4 by covalently targeting the active site selenocysteine residue U46 (5). Indeed, GPX4-expressing cells or affinity-purified GPX4 incubated with RSL3 or MI-2, but not MLT-985, caused a GPX4 shift indicative of covalent binding (5) (Fig. 2*B*). In MEFs, this shift was independent of MALT1 expression. Additionally, RSL3 and MI-2, but not MLT-985, inhibited GPX4 enzymatic activity in vitro (Fig. 2*C*). Covalent inhibitors can promote GPX4 degradation (6), and both MI-2 and RSL3 decreased GPX4 expression independently of MALT1 (Fig. 2*D*). MI-2, like RSL3, failed to induce ferroptosis in MEFs expressing the GPX4 U46C missense variant, which is more resistant to covalent GPX4 inhibitors due to the lower affinity of the chloroacetamide moiety toward cysteine (Fig. 2*E*) (7).

Our data failed to support a relevant role of MALT1 and Roquin-1 in counteracting or promoting ferroptosis. Instead, we demonstrate that MI-2 induces ferroptosis by directly inhibiting GPX4, independent of MALT1 or Roquin-1 (Fig. 2*F*). MI-2 lacks selectivity, targets MALT1 outside the active site, and couples to numerous cellular proteins (8). Thus, studies implicating MALT1 protease in ferroptosis based on MI-2 treatments need to be reevaluated (1, 9). Importantly, preclinical studies supporting targeting of MALT1 in chronic lymphocytic leukemia (CLL) have largely relied on MI-2 (10), which may have influenced the inclusion of CLL in clinical trials using allosteric MALT1 inhibitors (NCT03900598, NCT04876092, NCT05544019, NCT05515406, and NCT05618028). Collectively, we strongly advocate for excluding MI-2 from future analysis of MALT1 protease functions in physiological and pathological contexts.
